# Effects of solar radiation exposure on ischemic heart disease mortality: country-level spatial regression models

**DOI:** 10.1186/s41182-025-00813-6

**Published:** 2025-10-10

**Authors:** Haruka Kato, Satomi Ikeuchi, Susumu Tanimura

**Affiliations:** Department of Public Health Nursing, Mie University Graduate School of Medicine, 2-174 Edobashi, Tsu, Mie 514-8507 Japan

**Keywords:** Sunlight, Global solar radiation, Myocardial ischemia, Cardiovascular diseases, Spatial regression, Spatial analysis, Internationality, Global health

## Abstract

**Background:**

Previous studies have analyzed the association between sun exposure and ischemic heart disease (IHD). However, the association has not been assessed globally and may differ when adjusting for spatial dependency. This study aimed to clarify whether this global association remains even while incorporating spatial adjustment.

**Methods:**

The most recent age-adjusted IHD mortality data (per 100,000) by country (1987–2022) were obtained from the World Health Organization (WHO) database as the dependent variable. As the independent variable, global solar radiation (GSR) data (MJ/m^2^/day; mean of 1994–2018) were retrieved from the Global Solar Atlas, with values clipped to each capital’s location. Covariates included smoking prevalence, alcohol consumption, salt intake, gross domestic product, and health expenditure. To assess associations by sex, ordinary least squares (OLS) regression and three spatial regression models (spatial lag model, spatial error model, and spatial Durbin model) were applied. Additionally, an income-level stratified analysis was conducted. All analyses were performed with R version 4.5.0.

**Results:**

After listwise deletion of missing values, 94 countries remained. The mean (SD) IHD mortality rates for males and females were 96.5 (80.4) and 52.4 (48.0), respectively. The mean (SD) GSR was 15.9 (3.7). In the OLS model, GSR showed a significant negative association with IHD mortality (males: β =  − 8.82, p = 0.002; females: β =  − 6.31, p < 0.001). The spatial lag model was the best fit for both sexes, and the association persisted (males: β =  − 4.78, p = 0.041; females: β =  − 3.86, p = 0.005). Stratified analysis largely supported these findings. However, coefficients substantially decreased after spatial adjustment.

**Conclusions:**

Sun exposure retained a significant inverse association with ischemic heart disease mortality after adjusting for spatial dependency, although adjustment markedly reduced the strength of association. However, our results require careful interpretation due to several limitations in the study.

**Supplementary Information:**

The online version contains supplementary material available at 10.1186/s41182-025-00813-6.

## Background

Ischemic heart disease (IHD) is the leading cause of mortality worldwide, accounting for 13% of all global deaths. The World Health Organization (WHO) reported that since 2000, IHD mortality has increased more than that of any other cause [1]. Furthermore, IHD-related deaths rose from 1990 to 2021 [2], and the global mortality of IHD is expected to keep increasing until 2050 [3].

While excessive sun exposure is a known risk factor for skin cancer, appropriate exposure may prevent various health conditions, including cardiovascular disease, multiple sclerosis, type 2 diabetes, and rickets [4]. Previous studies have assessed the association between sun exposure and IHD [5–7], suggesting a preventive effect. However, these studies ignored spatial dependency, which distorts the association, and did not assess this relationship in a global setting with larger variation in sun exposure.

This study aimed to clarify whether this global association remains after accounting for spatial dependency. With changes in solar radiation related to climate change [8], understanding the effect of sunlight on human health, including IHD mortality, has become increasingly important. Sun exposure is a modifiable factor that can be increased through low-cost practices, such as sunbathing, and may serve as a feasible intervention in any country.

## Method

The latest available age-adjusted IHD mortality data (per 100,000 individuals; 1987–2022) were obtained for each country from the WHO database [9]. As an independent variable, global solar radiation (GSR) data (MJ/m^2^/day; mean of 1994–2018) were retrieved from the Global Solar Atlas [10] and clipped at each capital’s coordinates. Covariates included prevalence of current tobacco use (% of adults), total pure alcohol consumption (liters per capita), the natural logarithm of gross domestic product (GDP, International $ per capita), and current health expenditure (% of GDP), sourced from the World Development Indicator database [11]. Salt intake (g/day; 2019) data were collected from a 2023 WHO report [12]. These data were used without any transformation (except GDP) or standardization. The selection of covariates was based on established and available risk factors for IHD. All covariates except salt intake reflected 2020 values, corresponding to the median survey year for mortality. Furthermore, all analyses were conducted separately by sex due to potential sex differences in sun exposure behaviors, cardiovascular risk, and occupational patterns.

Ordinary least squares (OLS) regression model and three spatial regression models (spatial lag model [SLM], spatial error model [SEM], and spatial Durbin model [SDM]) were used to assess the associations by sex. Further subgroup analysis was stratified by income level because different settings by income level (e.g., variation in medical resources and data reliability) may modify the association. The best overall model was applied for subgroup analysis. The spatial weight matrix was created using the k-nearest neighbor method [13]: k = 7 for all, high-income, and upper-middle-income countries; and k = 6 for lower-middle-income countries due to a smaller sample size. Multicollinearity in the regression models was examined using variance inflation factors. Akaike’s information criterion (AIC) was used to determine the best model, and all analyses were conducted using R version 4.5.0.

## Results

**Fig. 1:**
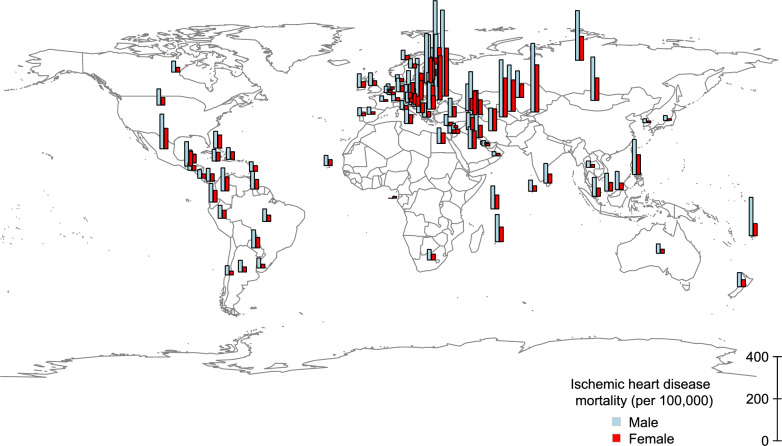
Distribution of ischemic heart disease. The most recent data for each country (1987–2022) from the WHO database [9]. Bar length represents ischemic heart disease mortality

**Fig. 2:**
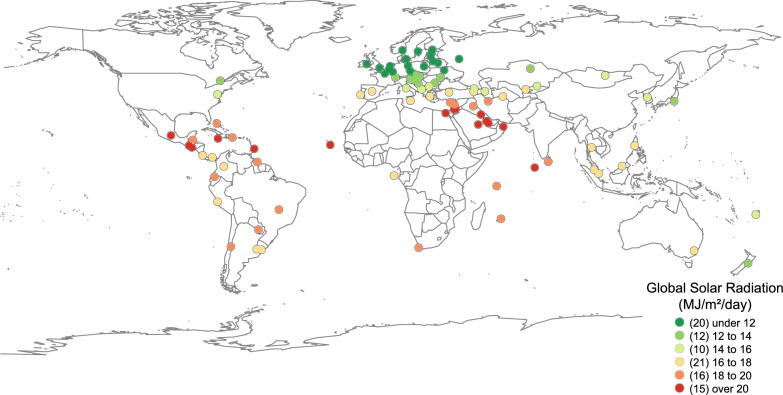
Distribution of global solar radiation. Values clipped from global data (mean of 1994–2018) [10] at each capital’s location. Numbers in parentheses in the legend indicate the number of countries per class division

After excluding records with missing values, 94 countries were analyzed. The regional and income-level classifications of precluded countries are shown in eTable 1. Descriptive statistics appear in Table 1. The IHD mortality, alcohol consumption, and smoking prevalence were higher among males than females (Table 1). Geographic distributions of IHD mortality and GSR are presented in Figs. 1 and 2. IHD mortality tended to be higher in Eastern Europe (Fig. 1). Higher GSR was observed around the solar equator, whereas lower GSR was concentrated in Europe (Fig. 2).

**Table 1:** Descriptive statistics (n = 94)

Variables		Mean	SD
GSR (MJ/m^2^/day)		15.9	3.7
GDP^a^		10.2	0.7
Health Expenditure (% of GDP)		7.9	2.7
Salt (g/day)		8.9	2.0
Males	IHD mortality (per 100,000)	96.5	80.4
	Alcohol (liters per capita)	10.5	6.3
	Smoking (%)	30.2	12.0
Females	IHD mortality (per 100,000)	52.4	48.0
	Alcohol (liters per capita)	2.8	1.8
	Smoking (%)	12.0	9.8

*GSR* global solar radiation, *GDP* gross domestic product, *IHD* ischemic heart disease, *SD* standard deviation

^a^GDP (International $) was transformed by the natural logarithm

In the OLS model, GSR showed a significant negative association with IHD mortality (Table 2). The spatial lag model was identified as the best model for both sexes, and the negative association persisted (Table 2). Spatial dependency was significant in this model (spatial parameter ρ in Table 2). Coefficients decreased substantially after adjusting for spatial dependency.

**Table 2:** Model comparison assessed the association between sun exposure and IHD mortality (n = 94)

	OLS		SLM		SEM		SDM	
	β	*P*	β	*P*	β	*P*	β	*P*
Males								
GSR (MJ/m^2^/day)	** − 8.82**	0.002	** − 4.78**	0.041	** − 6.48**	0.046	− 2.52	0.494
Alcohol (L per capita)	1.81	0.241	1.35	0.296	0.81	0.572	1.12	0.402
GDP^a^ (International $)	** − 45.81**	< 0.001	** − 36.62**	< 0.001	** − 33.72**	0.002	** − 31.34**	0.003
Smoking (%)	**1.66**	0.006	**1.21**	0.020	**1.78**	0.009	1.17	0.081
Salt (g /day)	− 1.48	0.664	− 1.06	0.711	0.57	0.883	0.25	0.948
Health Expenditure (% of GDP)	** − 8.38**	0.004	** − 5.11**	0.033	− 4.65	0.060	** − 6.03**	0.013
ρ			**0.62**	< 0.001			0.26	0.219
λ					**0.64**	< 0.001		
Adjusted R^2^	0.39							
F Statistic (df = 6; 87)	**10.86**	< 0.001						
AIC	1054.00		1036.11		1043.39		1036.65	
f^2^	0.75							
1 − β	1.00							
Females								
GSR (MJ/m^2^/day)	** − 6.31**	< 0.001	** − 3.86**	0.005	− 3.25	0.114	− 0.16	0.946
Alcohol (L per capita)	2.67	0.455	2.61	0.374	2.13	0.503	2.30	0.438
GDP^a^ (International $)	** − 32.72**	< 0.001	** − 26.63**	< 0.001	** − 26.12**	< 0.001	** − 24.10**	< 0.001
Smoking (%)	− 0.84	0.148	− 0.61	0.203	− 0.81	0.170	− 0.72	0.209
Salt (g /day)	0.05	0.982	− 0.26	0.884	0.51	0.831	0.18	0.937
Health Expenditure (% of GDP)	** − 4.91**	0.007	** − 2.90**	0.049	− 2.16	0.165	− 2.52	0.099
ρ			**0.66**	< 0.001			**0.41**	0.028
λ					**0.69**	< 0.001		
Adjusted R^2^	0.33							
F Statistic (df = 6; 87)	**8.53**	< 0.001						
AIC	965.85		944.49		952.79		946.28	
f^2^	0.59							
1 − β	1.00							

*OLS* ordinary least squares regression, *SLM* spatial lag model, *SEM* spatial error model, *SDM* spatial Durbin model, *GSR* global solar radiation, *GDP* gross domestic product, *AIC* Akaike's information criterion

Boldface denotes statistical significance

^a^GDP (International $) was transformed by the natural logarithm

Countries were stratified by the 2023 World Bank classification: high-income countries (n = 52), upper-middle-income countries (n = 33), and lower-middle-income countries (n = 9). Low-income countries were not included in this analysis due to missing data. In stratified analysis with the spatial lag model, significant negative associations were observed in upper-middle-income countries and lower-middle-income countries but not in high-income countries (see eTable 2 in the additional file). No multicollinearity was detected in any of the models.

## Discussion

After adjusting for spatial dependency, this study found a significant negative association between sun exposure and IHD mortality, confirmed in stratified analysis. The coefficients suggest that an increase of 1 MJ/m^2^/day in GSR could have prevented approximately 343,403 IHD deaths (3.8% of the total) in 2021. This association may indicate a potential preventive influence related to sufficient exposure to solar radiation. However, this result requires careful interpretation due to several limitations in this study.

Two possible mechanisms may contribute to this preventive effect: ultraviolet (UV) radiation and circadian rhythm adjustment. When human skin is exposed to UV-B radiation, vitamin D synthesis occurs [14]. Vitamin D may protect against IHD by regulating calcium homeostasis, supporting myocardial and smooth muscle function, and enhancing vascular endothelial function [15]. Additionally, UV-A radiation may exert vasodilatory effects [4, 16]. Though short-term, repeated daily exposure could maintain low blood pressure, possibly reducing IHD mortality risk. Sunlight also helps regulate the circadian rhythm [17]. Disruption of the rhythms is a direct cardiovascular disease risk factor [18] and an indirect risk factor through psychiatric disorders [19, 20]. Circadian rhythm dysregulation may be more common in low-sunlight regions, where higher IHD mortality was observed. Additionally, effects mediated by temperature and/or physical activity should be considered. In high-income countries, people may have sufficient medical resources and a better nutritional status, including vitamin D. Consequently, there might be no significant association for high-income countries.

This study has several limitations. First, as an ecological study, it is susceptible to ecological fallacies. To address this, further subnational or individual-level studies (e.g., cohort studies) are required. Second, the quality of IHD mortality and covariate data may differ across countries due to differences in healthcare systems, diagnostic criteria, and reporting systems. These differences may introduce information bias. Third, although outcomes reflect the most recent values, survey years varied among countries. Moreover, there was limited consistency among the years of the dependent, independent, and covariate variables, and such temporal mismatches may have introduced systematic bias. Fourth, missing data were not random, being more prevalent in African regions and in low- and lower-middle-income countries, which may have introduced selection bias. Fifth, GSR values were based on each country’s capital location, disregarding within-country heterogeneity. Thus, the GSR used may not comprehensively represent all areas within the country, particularly in geographically diverse or large countries. This may have led to non-differential exposure misclassification, potentially biasing associations toward the null. One potential improvement would be the use of population-weighted GSR, but tract-level census data were not available for most countries. Sixth, the covariates used in this study were limited because of procuring available open data. If other potential covariates (e.g., physical activity, obesity prevalence, healthcare access, air pollution, sun exposure behaviors, and occupation) were available, our results might have been different.

## Conclusions

Based on our global ecological analysis, sun exposure was significantly associated with lower ischemic heart disease mortality, even after adjusting for spatial dependency. However, spatial adjustment substantially reduced the strength of the association. These results require careful interpretation due to several limitations in the study.

## Supplementary Information

Supplementary material 1.

Supplementary material 2.

Supplementary material 3.

## Data Availability

The dataset supporting the conclusions of this article is included within an additional file of the article.

## Abbreviations

AIC: Akaike’s information criterion
GDP: Gross domestic product
GSR: Global solar radiation
IHD: Ischemic heart disease
OLS: Ordinary least squares
SDM: Spatial Durbin model
SEM: Spatial error model
SLM: Spatial lag model
UV: Ultraviolet
