# Supplementary material for: Effects of solar radiation exposure on ischemic heart disease mortality: country-level spatial regression models
Source: Trop Med Health. 2025 Oct 10;53:137. doi: 10.1186/s41182-025-00813-6 (PMC12512395; doi:10.1186/s41182-025-00813-6)
Supplement: Supplementary file 1 — Supplementary material 1. [file 41182_2025_813_MOESM1_ESM.pdf]

eTable 1. Income-level and region of included and excluded countries

Income level	Included countries		Excluded countries	
	n	(%)	n	(%)
High-income	52	(55.32%)	11	(11.11%)
Upper-middle-income	33	(35.11%)	20	(20.20%)
Lower-middle-income	9	(9.57%)	41	(41.41%)
Low-income	0	(0.00%)	26	(26.26%)
Others	0	(0.00%)	1[a]	(0.00%)
Total	94	(100.00%)	99	(100.00%)

Region	Included countries		Excluded countries	
	n	(%)	n	(%)
East Asia & Pacific	11	(11.70%)	19	(19.19%)
Europe & Central Asia	43	(45.74%)	9	(9.09%)
Latin America & Caribbean	19	(20.21%)	14	(14.14%)
Middle East & North Africa	12	(12.77%)	8	(8.08%)
North America	2	(2.13%)	0	(0.00%)
South Asia	2	(2.13%)	6	(6.06%)
Sub-Saharan Africa	5	(5.32%)	43	(43.43%)
Total	94	(100.00%)	99	(100.00%)

Included countries: countries included in this study, Excluded countries: countries excluded in this study due to missing value, Income level and region group were based on World Bank country classifications for 2023.

[a] Bolivarian Republic of Venezuela has been unclassified due to the unavailability of data.
