# Supplementary material for: Effects of solar radiation exposure on ischemic heart disease mortality: country-level spatial regression models
Source: Trop Med Health. 2025 Oct 10;53:137. doi: 10.1186/s41182-025-00813-6 (PMC12512395; doi:10.1186/s41182-025-00813-6)
Supplement: Supplementary file 2 — Supplementary material 2. [file 41182_2025_813_MOESM2_ESM.pdf]

eTable 2. Comparison between income level groups

	Males' ordinary least squares regression model							
	All countries (n = 94)		High-income countries (n = 52)		Upper-middle-income countries (n = 33)		Lower-middle-income countries (n = 9)	
	β	*P*	β	*P*	β	*P*	β	*P*
GSR (MJ/m^2/day)	**−8.82**	0.002	−4.18	0.096	**−24.21**	0.001	−26.12	0.367
Alcohol (liters per capita)	1.81	0.241	1.10	0.492	1.96	0.499	−0.73	0.972
GDP[a]	**−45.81**	<0.001	**−38.21**	0.026	−34.12	0.475	−105.80	0.471
Smoking (%)	**1.66**	0.006	**2.37**	0.002	−0.55	0.640	3.06	0.471
Salt (g /day)	−1.48	0.664	0.86	0.767	−14.19	0.073	4.65	0.901
Health Expenditure (% of GDP)	**−8.38**	0.004	**−6.12**	0.011	1.77	0.802	−22.23	0.559
Adjusted R^2	0.39		0.46		0.44		0.30	
F Statistic	**10.86**	<0.001	**8.24**	<0.001	**5.15**	0.001	1.58	0.437
AIC	1054.00		538.76		385.70		108.81	
f^2	0.75		1.10		1.19		4.74	
1 − β	1.00		1.00		1.00		0.62	
	**Males' spatial lag model**							
	**All countries (n = 94)**		**High-income countries (n = 52)**		**Upper-middle-income countries (n = 33)**		**Lower-middle-income countries (n = 9)**	
	β	*P*	β	*P*	β	*P*	β	*P*
GSR (MJ/m^2/day)	**−4.78**	0.041	−3.73	0.111	**−25.15**	<0.001	**−27.36**	0.009
Alcohol (liters per capita)	1.35	0.296	1.10	0.454	1.71	0.508	−1.20	0.888
GDP[a]	**−36.62**	<0.001	**−35.04**	0.023	−35.16	0.399	**−114.36**	0.043
Smoking (%)	**1.21**	0.020	**2.24**	0.001	−0.38	0.727	2.90	0.074
Salt (g /day)	−1.06	0.711	0.55	0.839	**−14.08**	0.036	1.96	0.900
Health Expenditure (% of GDP)	**−5.11**	0.033	**−5.97**	0.005	1.67	0.787	−23.72	0.111
ρ	**0.62**	<0.001	0.14	0.515	−0.09	0.787	0.34	0.637
AIC	1036.11		540.34		387.63		110.58	

(Continued on next page)

eTable 2 (continued)

	Females' ordinal least squares regression							
	**All countries (n = 94)**		**High-income countries (n = 52)**		**Upper-middle-income countries (n = 33)**		**Lower-middle-income countries (n = 9)**	
	β	*P*	β	*P*	β	*P*	β	*P*
GSR (MJ/m²/day)	**−6.31**	<0.001	−2.04	0.196	**−13.13**	0.001	−17.56	0.437
Alcohol (liters per capita)	2.67	0.455	3.57	0.333	3.56	0.573	−36.91	0.428
GDP[a]	**−32.72**	<0.001	**−26.69**	0.011	−13.91	0.643	−104.32	0.467
Smoking (%)	−0.84	0.148	−0.30	0.560	−2.57	0.117	1.05	0.834
Salt (g /day)	0.05	0.982	2.43	0.204	−9.02	0.061	3.51	0.893
Health Expenditure (% of GDP)	**−4.91**	0.007	**−4.16**	0.006	2.67	0.557	−23.94	0.496
Adjusted R^2	0.33		0.28		0.37		0.24	
F Statistic	**8.53**	<0.001	**4.23**	0.002	**4.09**	0.005	1.42	0.468
AIC	965.85		491.81		351.92		102.87	
f^2	0.59		0.56		0.94		4.27	
1 − β	1.00		0.99		0.99		0.57	

	Females' spatial lag model							
	**All countries (n = 94)**		**High-income countries (n = 52)**		**Upper-middle-income countries (n = 33)**		**Lower-middle-income countries (n = 9)**	
	β	*P*	β	*P*	β	*P*	β	*P*
GSR (MJ/m²/day)	**−3.86**	0.005	−1.77	0.219	**−12.74**	<0.001	**−19.38**	0.021
Alcohol (liters per capita)	2.61	0.374	3.62	0.281	3.86	0.496	**−36.29**	0.033
GDP[a]	**−26.63**	<0.001	**−23.68**	0.011	−14.08	0.593	**−108.01**	0.042
Smoking (%)	−0.61	0.203	−0.37	0.433	−2.56	0.068	0.81	0.687
Salt (g /day)	−0.26	0.884	2.00	0.260	**−9.01**	0.027	0.67	0.949
Health Expenditure (% of GDP)	−2.90	0.049	**−3.94**	0.003	2.64	0.509	−24.18	0.065
ρ	**0.66**	<0.001	0.22	0.326	0.04	0.874	0.43	0.519
AIC	944.49		492.85		353.89		104.45	

Abbreviation: GSR, global solar radiation; GDP, gross domestic product; AIC, Akaike's Information Criterion.

[a] GDP (International $) was transformed by the natural logarithm. Note: Boldface indicates statistical significance.
