# Supplementary material for: Effects of solar radiation exposure on ischemic heart disease mortality: country-level spatial regression models
Source: Trop Med Health. 2025 Oct 10;53:137. doi: 10.1186/s41182-025-00813-6 (PMC12512395; doi:10.1186/s41182-025-00813-6)
Supplement: Supplementary file 3 — Supplementary material 3. [file 41182_2025_813_MOESM3_ESM.pdf]

eTable 3: Data used in the current study

	Country	income	GSR	GDP	salt	HE	Male IHD	Male alcohol	Male smoking	Female IHD	Female alcohol	Female smoking
1	Albania	U	15.65	9.58	12.80	7.50	74.78	7.37	38.80	46.88	1.59	6.00
2	Argentina	U	17.27	10.02	9.20	10.45	55.76	12.59	29.40	24.47	3.71	19.60
3	Armenia	U	16.65	9.60	8.80	12.24	203.53	7.49	49.40	112.18	1.83	1.50
4	Australia	H	17.26	10.90	7.40	10.66	44.45	15.92	15.60	19.42	4.83	11.50
5	Austria	H	11.94	10.98	8.60	11.32	68.85	18.09	27.70	31.94	5.22	25.00
6	Azerbaijan	U	14.92	9.63	8.90	5.85	114.25	4.44	47.90	69.77	0.68	0.10
7	Belgium	H	10.61	10.94	8.30	11.26	30.80	14.83	25.80	11.67	4.25	21.00
8	Bulgaria	H	14.07	10.16	12.90	8.48	133.34	19.03	40.90	64.28	4.77	37.10
9	Bahrain	H	20.89	10.89	6.60	4.72	16.01	1.78	25.30	9.82	0.26	4.50
10	Bahamas, The	H	19.04	10.17	7.30	7.59	79.76	6.03	18.80	62.18	1.52	2.40
11	Bosnia and Herzegovina	U	13.20	9.70	12.80	9.70	84.29	9.90	42.00	48.65	2.03	28.00
12	Belarus	U	10.43	10.12	7.00	6.41	408.99	19.44	47.40	185.89	5.21	13.50
13	Belize	U	18.37	9.17	7.30	5.35	61.54	5.85	15.10	41.04	1.48	1.80
14	Brazil	U	19.96	9.69	9.00	9.62	59.84	12.37	16.20	30.43	3.27	9.40
15	Barbados	H	21.01	9.67	6.80	7.27	45.96	13.64	15.00	27.87	3.35	1.90
16	Brunei	H	17.95	11.15	11.40	2.45	86.18	0.82	30.00	31.47	0.17	2.30
17	Canada	H	13.18	10.79	9.10	13.02	52.62	15.59	15.30	23.30	4.31	10.70
18	Switzerland	H	12.69	11.20	8.40	12.00	35.16	15.68	28.10	14.93	4.61	22.90
19	Chile	H	19.85	10.14	9.40	9.69	43.86	11.77	31.60	18.23	3.46	26.80
20	Colombia	U	17.09	9.65	12.00	8.71	109.30	6.98	12.40	66.61	1.63	4.60
21	Cabo Verde	L	21.05	8.83	6.90	6.63	48.44	9.62	17.30	28.51	2.49	5.40
22	Costa Rica	U	17.65	10.00	10.20	7.83	40.34	5.54	12.90	19.83	1.28	4.60
23	Cyprus	H	18.99	10.68	8.50	8.76	52.40	10.07	47.00	15.03	2.66	23.20
24	Czechia	H	11.09	10.71	13.00	9.16	114.23	19.05	35.00	57.76	5.25	26.40
25	Germany	H	10.53	10.98	8.70	12.69	64.93	18.63	24.10	28.24	5.30	19.90
26	Denmark	H	10.16	11.05	8.20	10.72	31.72	14.77	17.80	13.21	4.33	17.10
27	Dominican Republic	U	19.91	9.86	7.30	4.94	56.22	10.31	14.60	36.46	2.69	6.50
28	Ecuador	U	19.31	9.35	9.00	8.66	88.54	3.95	18.40	55.56	1.15	4.20
29	Egypt	L	20.72	9.63	6.40	4.15	74.45	0.23	48.10	50.38	0.03	0.40
30	Spain	H	17.14	10.58	8.20	10.73	34.04	14.52	28.60	11.63	4.09	26.70
31	Estonia	H	9.88	10.61	5.70	7.58	90.06	17.40	36.30	31.57	4.82	23.00
32	Finland	H	9.72	10.88	8.40	9.63	77.75	14.25	26.90	29.24	4.10	16.30
33	Fiji	U	15.08	9.33	7.40	5.29	183.02	5.97	35.60	57.77	1.19	10.50
34	France	H	11.46	10.81	7.60	12.07	26.14	16.48	34.90	8.52	4.68	31.90
35	United Kingdom	H	10.16	10.78	7.10	11.97	60.95	16.83	17.30	23.26	4.88	13.50
36	Georgia	U	14.54	9.73	8.80	8.34	126.60	24.51	56.30	57.47	5.87	7.10
37	Greece	H	17.26	10.29	8.30	9.52	66.82	9.29	36.50	26.31	2.51	30.50
38	Guatemala	U	20.86	9.30	10.20	6.46	116.49	2.53	20.10	70.39	0.59	1.60
39	Guyana	H	19.39	9.73	7.30	5.42	84.71	8.23	21.70	45.73	2.09	2.40
40	Croatia	H	12.73	10.36	12.90	7.65	96.75	12.35	37.60	50.96	3.27	36.10
41	Hungary	H	12.48	10.46	14.30	7.29	169.51	16.12	35.80	93.38	4.36	27.80
42	Ireland	H	9.43	11.50	7.30	7.06	64.90	16.60	22.50	27.38	5.15	19.00
43	Iran	U	18.97	9.55	6.30	5.40	108.10	0.90	24.10	104.25	0.10	3.10
44	Iraq	U	19.30	9.27	6.40	5.76	81.01	0.42	35.10	57.10	0.06	1.80
45	Israel	H	20.62	10.62	8.50	7.70	34.42	4.53	28.90	15.14	1.21	13.50
46	Italy	H	15.80	10.70	9.70	9.62	41.08	11.15	26.60	18.64	3.07	19.50
47	Jamaica	U	20.53	9.10	7.30	6.63	55.00	5.03	15.00	41.74	1.22	3.80
48	Jordan	L	20.98	9.17	6.40	7.12	46.85	0.39	56.80	21.05	0.05	12.80
49	Japan	H	13.99	10.66	10.20	11.21	22.86	10.27	30.10	7.68	2.76	10.00
50	Kazakhstan	U	12.41	10.28	8.90	3.75	126.40	8.06	39.60	64.07	1.43	6.70
51	Kyrgyzstan	L	15.33	8.55	9.00	4.95	325.73	6.40	48.00	224.40	1.05	2.80
52	Korea, South	H	14.24	10.72	12.30	8.36	18.27	12.18	35.70	8.16	3.43	5.90
53	Kuwait	H	20.16	10.63	8.30	6.23	119.06	0.00	33.50	58.09	0.00	2.20
54	Lebanon	L	18.70	9.70	6.20	7.58	22.29	2.28	47.50	11.25	0.34	28.90
55	Sri Lanka	L	19.28	9.47	10.70	4.06	93.37	4.98	41.40	43.57	1.05	2.60
56	Lithuania	H	10.08	10.63	7.40	7.47	233.72	19.82	42.10	118.58	5.50	21.80
57	Luxembourg	H	11.07	11.71	8.50	5.76	24.14	16.50	22.40	11.63	5.10	19.80
58	Latvia	H	10.14	10.40	7.60	7.29	213.61	21.35	50.30	90.04	5.87	23.70
59	Moldova	U	12.92	9.51	7.10	7.00	312.63	18.09	51.70	210.27	4.94	6.20
60	Maldives	U	20.11	9.50	10.30	11.34	53.09	2.32	44.40	26.27	0.27	6.00
61	Mexico	U	21.03	9.87	8.80	6.05	165.07	7.90	19.90	97.93	1.90	6.20
62	Malta	H	17.95	10.82	9.60	10.60	80.91	10.84	26.40	43.06	3.11	21.60
63	Mongolia	U	15.21	9.52	9.00	4.67	206.48	12.35	51.70	107.42	3.22	7.10
64	Mauritius	U	18.81	9.97	10.80	6.10	129.63	11.41	37.30	70.14	2.47	3.00
65	Malaysia	U	16.37	10.22	10.50	4.08	84.94	1.26	43.80	41.40	0.20	1.10

Continued on next page

eTable 3: Data used in the current study

	Country	income	GSR	GDP	salt	HE	Male			Female		
							IHD	alcohol	smoking	IHD	alcohol	smoking
66	Netherlands	H	10.37	11.04	8.20	11.22	25.98	13.59	24.40	11.43	3.95	19.90
67	Norway	H	9.35	11.11	7.80	11.42	44.76	11.38	17.00	20.11	3.35	15.40
68	New Zealand	H	13.67	10.72	8.00	10.06	67.46	15.53	15.00	33.42	4.70	12.30
69	Oman	H	21.53	10.47	7.10	5.12	19.91	0.72	15.50	12.18	0.10	0.40
70	Panama	H	17.32	10.20	10.20	10.22	61.45	7.55	7.70	35.31	1.82	2.20
71	Peru	U	16.80	9.44	9.00	6.36	61.82	10.04	13.20	38.41	2.99	3.00
72	Philippines	L	16.88	9.02	10.40	5.16	166.51	9.10	39.30	93.97	2.18	6.50
73	Poland	H	10.75	10.51	11.10	6.50	109.36	18.74	27.90	56.70	5.16	20.10
74	Portugal	H	17.07	10.49	8.90	10.55	38.38	14.40	30.50	14.21	4.06	20.20
75	Paraguay	U	18.29	9.57	9.10	7.68	85.81	8.88	18.60	50.11	2.37	4.40
76	Qatar	H	20.98	11.32	7.20	3.82	25.53	1.29	21.70	12.62	0.23	1.90
77	Romania	H	13.60	10.45	12.90	6.23	143.89	27.03	35.90	80.61	7.42	20.00
78	Russia	H	10.22	10.36	9.70	8.04	236.59	18.14	40.80	113.94	4.20	12.80
79	Saudi Arabia	H	21.97	10.72	6.70	6.65	103.19	0.00	26.50	84.44	0.00	2.00
80	Singapore	H	16.18	11.53	11.50	5.62	89.58	2.76	28.00	39.38	0.76	5.00
81	El Salvador	U	21.03	9.15	10.10	8.95	34.74	5.35	14.10	21.52	1.25	1.70
82	Sao Tome and Principe	L	17.73	8.55	6.90	7.32	0.00	7.61	10.10	8.64	2.00	1.30
83	Slovakia	H	12.21	10.47	12.80	7.13	183.11	17.05	37.40	111.59	4.68	25.60
84	Slovenia	H	12.67	10.64	13.00	9.43	47.99	16.40	24.40	19.21	4.42	19.60
85	Sweden	H	9.80	10.96	8.20	11.33	44.49	14.82	29.80	19.60	4.30	18.20
86	Seychelles	H	18.89	10.34	7.50	6.03	109.68	15.67	34.00	66.43	3.95	6.40
87	Thailand	U	17.65	9.86	10.80	4.36	30.68	13.29	41.30	14.32	3.08	2.90
88	Turkmenistan	U	16.60	9.62	9.00	5.57	271.69	4.60	10.60	185.11	0.74	0.40
89	Turkey	U	16.34	10.26	5.30	4.62	88.70	2.99	42.10	49.04	0.41	19.20
90	Ukraine	U	11.54	9.65	7.00	7.56	408.95	15.50	40.00	228.44	4.05	11.50
91	Uruguay	H	16.93	10.16	9.40	9.07	47.76	8.97	24.40	18.05	2.55	18.50
92	United States	H	15.05	11.07	8.90	18.81	76.93	15.54	28.40	35.85	4.43	17.50
93	Uzbekistan	L	16.48	9.04	9.00	6.71	220.32	3.67	34.00	149.62	0.57	1.10
94	South Africa	U	18.92	9.45	6.50	9.01	50.09	12.41	34.00	28.11	2.31	6.50

Abbreviation: L, lower-middle-income country; U, upper-middle-income country; H, high-income country; GSR, global solar radiation (MJ/m^2/day); GDP, the natural logarithm of gross domestic product (International $ per capita); HE, health expenditure (% of GDP); IHD, ischemic heart disease mortality (age-adjusted, per 100,000)
Note: the IHD mortality data (1987–2022) were obtained from World Health Organization (WHO) database [9]. GSR (mean of 1994–2018) was retrieved from Global Solar Atlas [10]. The prevalence of current tobacco use (% of adults), total pure alcohol consumption (L per capita), GDP, and current HE in 2020 were obtained from the World Development Indicator database [11] using WDI package in R. Salt intake (g/day) data was retrieved from the WHO report [12]. Income groups were based on World Bank country classifications for 2023. See references in main text for these citation numbers.
